# Psychiatric manifestations of huntington’s disease: Two case reports

**DOI:** 10.1192/j.eurpsy.2021.1093

**Published:** 2021-08-13

**Authors:** D. Göy, Ö. Şahmelikoğlu Onur, N. Karamustafalıoğlu

**Affiliations:** Psychiatry, Bakirkoy Research & Training Hospital for Psychiatry, Neurology and Neurosurgery, İstanbul, Turkey

**Keywords:** Huntington’s Disease, bipolar disorder, Depression, Movement Disorder

## Abstract

**Introduction:**

Huntington’s disease (HD) is a progressive neuropsychiatric and degenerative disorder that shows an autosomal dominant inheritance pattern. The principal symptoms of HD are progressive movement disorders, cognitive deterioration, dementia, and certain psychiatric manifestations, which may occur in many different forms, such as depression, psychosis, personality changes.

**Objectives:**

In this presentation, two female HD patients with psychotic components complicated with suicidal and homicidal thoughts will be reported to better illustrate psychiatric components of HD.

**Methods:**

Hospitalization records of these two patients with genetically verified HD diagnosis indicated that their psychiatric health precipitously deteriorated in the last decade.

**Results:**

While the first patient suffered from severe depression, anxiety, suicidality; persecution ideas, suspiciousness, hostility were noted more prominently in the latter. Moreover, both cases had a positive family history for psychiatric diseases which is one of the hallmarks of HD. Anti-psychotic drug olanzapine, which has minor side-effects on EPS, was found to be highly effective on our HD patients, alleviating the behavioral and psychiatric symptoms of the disease.

**Conclusions:**

In conclusion, HD should be one of the differential diagnoses if patients with psychiatric complaints have accompanying neurological findings such as movement disorders and impaired memory, and great attention should be paid to the extrapyramidal system (EPS) sensitivity of the chosen treatment regime when treating the HD patients,

